# Why healthcare providers are not vaccinated? A qualitative study during the COVID-19 pandemic in Iran

**DOI:** 10.1186/s12875-023-02166-7

**Published:** 2023-10-13

**Authors:** Fatemeh Kokabisaghi, Fatemeh Akhtar, Ali Taghipour, Javad Javan-Noughabi, Javad Moghri, Seyed Saeed Tabatabaee

**Affiliations:** 1 Social Determinants of Health Research Center, Mashhad University of Medical Sciences, Mashhad, Iran; 2 Department of Health Economics and Management Sciences, School of Health, Mashhad University of Medical Sciences, Mashhad, Iran; 3 Department of Epidemiology & Biostatistics, School of Health, Mashhad University of Medical Sciences, Mashhad, Iran

**Keywords:** Refusal, Acceptability, Willingness, Healthcare provider, COVID-19 vaccine

## Abstract

**Background:**

Vaccination has been effective in controlling contagious diseases, especially among high-risk groups such as medical staff. Their unwillingness to be vaccinated might adversely affect individual and public health. This study aimed to explore the factors related to the refusal of COVID-19 vaccines among health service providers.

**Methods:**

A qualitative study was conducted on 28 healthcare providers in Mashhad, Northeast of Iran from March to June 2022. The method of data collection was face-to-face interviews. The purposive method was used for sampling. Data collection continued until the saturation was reached. To analyze the data, the content analysis method was applied, and Maxqda (version 10) software was used.

**Results:**

By analyzing interview transcripts, six themes and ten sub-themes were extracted. Factors that explained employees’ reluctance to be vaccinated against COVID-19 were the opinion of peers, lack of trust in vaccines, fear of vaccination, mistrust to the government and health authorities, low perceived risk of coronavirus disease, and the contradictions of traditional and modern medicine in their approach to controlling the disease.

**Conclusions:**

Among healthcare workers, concerns about the side effects of vaccines were the most influential factors in refusing vaccination. Providing reliable information about vaccines and their safety is key to increasing the trust of health workers in vaccination and facilitating its acceptance.

**Supplementary Information:**

The online version contains supplementary material available at 10.1186/s12875-023-02166-7.

## Background

In recent years, the COVID-19 pandemic has brought numerous health problems, medical expenses and, political, social, and economic complications [1, 2]. Vaccination is the most effective way to control the disease and reduce mortality, especially among high-risk groups such as medical staff [3]. The availability of COVID-19 vaccines does not lead to their acceptance [4]. Although, the success of vaccination depends on the general acceptance of the vaccines, studies show that public trust in vaccines is decreasing [5]. Hesitancy in vaccination has become a global issue recently. According to the World Health Organization (WHO), it was one of the top ten threats to global health in 2019 [6].

During the COVID-19 pandemic, healthcare workers have been at high risk of infection, and vaccine hesitancy could affect their lives. According to Amnesty International 2021, one healthcare worker died every 30 min, and, more than 17,000 died worldwide from COVID-19 in one year [7]. In addition to the possibility of spreading the disease to their families and patients, the doubt of health professionals in vaccines can lead to the doubt of the general public [8]. The study by Wei and Fu’s (2019) aimed to discover the factors influencing vaccine acceptance, showed that the doubt about vaccination among the health and medical staff and the lack of knowledge about vaccines may weaken people’s trust in vaccines [9]. Therefore, WHO considered medical staff a high-priority group for COVID-19 vaccination [10].

Almost all over the world, the acceptance rate of COVID-19 vaccines has been far from its desired value among healthcare staff [11]. Several studies showed that not all healthcare workers were ready to accept the vaccine [12–14]. For example, a study in the Democratic Republic of Congo showed that only 28% of healthcare workers were willing to get the COVID-19 vaccine [15]. A Study in Greece showed that a small proportion of healthcare workers were willing to be vaccinated against COVID-19 [16]. The reluctance of nurses to get the COVID-19 vaccine has also been reported in China [17].

Psychological, social, and cultural factors affected the acceptance of vaccination [18–21]. Some of the reasons for the hesitancy in acquiring COVID-19 vaccines are linked to concerns about vaccine safety and side effects, mainly because the development and approval processes of these vaccines were very fast [17, 22, 23]. Vaccine acceptance can be influenced by people’s health beliefs, perceived risk of vaccination and disease and its severity, the perceived need for a vaccine, self-efficacy, and the risks and benefits of vaccination [24]. According to Li’s study, healthcare staff generally did not have a positive attitude toward COVID-19 vaccination. Women and nurses were more skeptical about the vaccine [25]. Concerns for the safety, efficiency, and effectiveness and mistrust to the government were expressed as barriers to accepting the vaccine [26]. In addition, factors such as misinformation, fear of vaccine side effects, difficulties in access to vaccines, and unfavorable previous vaccination history could make people hesitate to get the COVID-19 vaccine [5, 27–30].

Because of the high importance of coronavirus vaccination in preventing and controlling the disease, the refusal of healthcare workers can adversely affect public attitudes and beliefs [29].

Few studies investigated COVID-19 vaccine reluctance in Iran; none focused on the reasons behind it, particularly among healthcare professionals. Four studies investigated Iranians’ logic for not acquiring the vaccine [31–34]. Among them, two examined the vaccine acceptance and two the attitudes of the population about the COVID-19 vaccination [35–38]. In addition, a review has been conducted on the willingness and unwillingness to acquire COVID-19 vaccination at the international level.

Since healthcare professionals are the trusted sources of information on public health issues, including COVID-19 vaccines, discovering their reasons for vaccine reluctance can help to prevent and solve this issue. This study aimed to determine the factors related to the refusal of COVID-19 vaccines among healthcare workers in Iran.

## Methods

This qualitative research has been carried out by applying the conventional content analysis method. In Iran, vaccination against COVID-19 started in February 2021, at the same time as the fourth Corona outbreak wave. This study was conducted between March to June 2022 and after the sixth wave of Corona.

The population of the study was healthcare workers in Mashhad City that have been refused to get the COVID-19 vaccine. The inclusion criteria were at least six months of work experience in healthcare settings and the willingness to participate in the study. The exclusion criterion was the unwillingness to continue cooperation during the study. A total of 28 healthcare staff of hospitals and health centers affiliated with Mashhad University of Medical Sciences were selected using purposive sampling to participate in the study.

In the first instance, the researchers got a list of healthcare workers reluctant to acquire the vaccine from the authorities. Then, clinical staff were selected from the list, and administrative employees were excluded. The researchers tried to have the most variety in demographic characteristics such as gender, age, marital status, job, education, place of work, years of work experience, and type of employment contract. The data collection method was face-to-face interviews. In order to formulate the questions, preliminary interviews were conducted in an in-depth and unstructured manner. These questions were completed during the research phases and turned into a semi-structured questionnaire. There was a supplementary file including a questionnaire/interview guide.

The interviews were conducted at the participants’ workplace and lasted for an average of 30 min. The interviews started with a general question, “why don’t you get the vaccine?” and then, the discussion was directed to the details with the questions such as “could you explain more? Do you mean that…?” By creating a friendly atmosphere and paying attention to the person’s feelings, the interviewees were allowed to continue talking as long as their talk was related to the topic. All interviews were fully recorded and then transcribed. In addition, a summary of the important issues raised during the interviews was provided to the interviewees to ensure mutual understanding. Data collection continued until data saturation was reached, that is when the participants raised no new content.

The rigor and trustworthiness of the data (credibility, dependability, confirmability, and transferability) were assessed using the Guba and Lincoln method [39].

Transferability refers to the extent to which the results can be applied to different contexts. To ensure about transferability, the researchers provided comprehensive details about the research setting, participant experiences, data analysis, and study findings to the readers enabling them to assess the applicability of the findings [39].

To ensure credibility, the study team conducted regular debriefing sessions with the data collectors. They discussed their experiences with the study team members to address any biases that arose during the interview process. Also, techniques such as active listening, extended engagement with the data, immersive data analysis, and triangulation of data sources and investigators were employed. Additionally, sampling was conducted with a maximum variation of age, gender, marital status, job, education, place of work, years of work experience, and type of employment contract [39].

To ensure the dependability of the findings, the researchers documented and kept a record of analysis for audit trailing. The confirmability of the study findings was ensured by peer-checking and member-checking techniques [39].

The data was analyzed by MAXQDA (version 10). Data was analyzed using the conventional content analysis method suggested by Graneheim and Lundman [40]. All of the interviews were conducted by the corresponding author (SST) and another author (FA). In the next step, two authors (FKS and JJN) transcribed and analyzed the data independently. The meaning units were identified, and the initial codes were extracted after immersing the data. Next, the initial codes were categorized into subcategories. By considering the relationships, similarities, and differences, categories were determined. Disagreements between two researchers regarding codes or categories were resolved by comments from the other authors (AT and JM). The output of this stage was the general framework of factors related to the refusal of health workers to get vaccinated against COVID-19.

## Results

As shown in Tables 1, 28 healthcare providers (17 working in hospitals and 11 in health centers) were interviewed. The majority of participants were women (n = 21; 25%), married (n = 25; 89.3%), had permanent employment contract (n = 20; 71.43%), and held bachelor’s degrees (n = 17; 60.7%).

**Table 1:** Demographic information of study participants

Variable	Levels	Number (percentage)
Gender	woman	21(75%)
	Man	7(52%)
Marital status	Married	25(89.3%)
	Single	3(10.7%)
Type of employment	Permanent	21(75%)
	Temporary	7(25%)
Education	Associate degree	3(10.7%)
	Bachelor	17(60.7%)
	Master	7(25%)
	MD	1(3.6%)
Job	Nurse	4(14.3)
	Midwife	4(14.3)
	Pharmacist	1(3.6)
	Healthcare Worker	5(17.9)
	Psychologist	2(7.1)
	Health information technician	2(7.1)
	Radiology technician	1(3.6)
	Nutritionist	1(3.6)
	Registration staff	3(10.7)
	Community health worker	1(3.6)
	Nurse aid	2(7.1)
	Department Secretary	1(3.6)
	Operation room nurse	1(3.6)
**Variable**	**mean**	**Standard deviation**
Age	42.29	6.87
Work experience	14.91	6.7

Regarding the reluctance of health workers to get vaccinated against COVID-19, in general, six themes, including the influence of peers, lack of trust in the vaccine, fear of vaccination, mistrust to the government and health authorities, low perceived risk for disease, and contradiction of traditional and modern medicine in their approach to controlling Coronavirus disease were found. These results are shown in Table 2.

**Table 2:** Detailed information of the participants

Code	Gender	Age	Marital status	Job	Education	Place of work	Years of work experience	Type of employment	Interview duration (minutes)
P1	woman	41	married	Nurse	bachelor	hospital	15	permanent	18
P2	woman	43	married	Psychologist	bachelor	hospital	10	permanent	25
P3	woman	41	single	Midwife	master	health center	12	permanent	21
P4	woman	40	single	IT technician	bachelor	hospital	14	permanent	20
P5	woman	29	married	IT technician	bachelor	hospital	15	permanent	19
P6	man	43	married	Radiology technician	bachelor	hospital	20	permanent	18
P7	woman	54	married	Nutritionist	Master	health center	25	permanent	31
P8	woman	46	married	Health education expert	master	health center	18	temporary	26
P9	woman	39	single	Family health worker	bachelor	health center	14	permanent	38
P10	woman	43	married	Family health worker	bachelor	health center	13	permanent	22
P11	woman	51	married	Diseases expert	bachelor	health center	24	permanent	17
P12	woman	39	married	Psychologist	master	hospital	13	permanent	23
P13	woman	39	married	Nurse	Master	hospital	12	permanent	25
P14	woman	40	married	Midwife	Master	health center	13	temporary	24
P15	woman	38	married	Midwife	bachelor	health center	3	temporary	32
P16	man	57	married	Pharmacist	MD	hospital	27	permanent	29
P17	man	36	married	Registration staff	bachelor	hospital	3	temporary	28
P18	man	40	married	Community health worker	associate	health center	25	permanent	18
P19	man	41	married	Nurse aid	associate	hospital	7	temporary	24
P20	man	39	married	Nurse	bachelor	hospital	14	temporary	31
P21	woman	33	married	Secretory	bachelor	hospital	10.5	permanent	29
P22	woman	39	married	Nurse	master	hospital	13	temporary	17
P23	woman	36	married	Nurse aid	associate	hospital	10	permanent	13
P24	man	40	married	Operation room nurse	bachelor	hospital	5	permanent	26
P25	woman	53	married	Midwife	bachelor	health center	20	permanent	22
P26	woman	54	married	Registration staff	bachelor	hospital	20	permanent	24
P27	woman	39	married	Registration staff	bachelor	hospital	15	permanent	26
P28	woman	54	married	Family health worker	bachelor	health center	27	permanent	22

The details about the themes and subthemes and the statements of the study participants about COVID-19 vaccine refusal are provided in the following paragraphs. Demographic information of the participants is shown in Table 3.

**Table 3:** Factors related to the refusal of COVID-19 vaccines among healthcare providers

Themes	Ssub-themes	Items
Peer influence	Belief in the advice of colleagues	Healthcare workers’ advice
		Doctor’s advice
Lack of trust in vaccines	Concerns about vaccine side effects	Having a chronic condition or specific disease
		Pregnancy and fear of harm to the fetus
		Breastfeeding and fear of harming the baby
		Fear of immediate side effects of the vaccine
		Fear of vaccine complications in the future
	Skepticism in the ineffectiveness of vaccination	Corona infection in vaccinated people
	Lack of transparency in vaccine-related information and statistics	Contradictory information provided by the government and social media
		The unknown nature of vaccines
Fear of vaccination	Having an unpleasant experience with the injection	Fear of needles and injections
Lack of trust in the government and health officials	Inappropriate policies	Mandatory vaccination
	Lack of access to reliable vaccines	Failure to import reliable European and American vaccines
		Unavailability of vaccines
Not feeling the need for a vaccine	natural immunity	Previously infected by Coronavirus
	Confidence in self-care	Willingness to follow other health protocols
	Low perceived risk of getting infected with Coronavirus	Not being afraid of coronavirus complications
Contrasting traditional and modern medicine	Trust in traditional medicine	Recommendations about traditional medicines

### Peer influence

Some participants decided not to get the vaccine after consulting with their colleagues. In this regard, a participant said: “I asked a few colleagues and they believed that it is better not to get the vaccine.“ (P 19) and “I know several experts who do not have positive opinions about the vaccine and are not willing to get vaccinated. After consulting with them, I was convinced that I should not get vaccinated and I was very scared”. (P12)

Some participants who suffered a chronic disease or condition decided not to get vaccinated based on their doctor’s advice. One of them stated: “I have epilepsy. When I consulted with my doctor, he said that the vaccine might adversely affect the brain, so it’s better if I don’t get it.“ (P13) " I didn’t get the vaccine because of its complications; I used to suffer from severe allergies and hives for two or three months. The doctor advised me not to take the vaccine.” (P27) Another said: “I was infected with Coronavirus and some of my symptoms remained. For example, my sense of smell was gone. I consulted a doctor and he told me not to get vaccinated.“ (P17).

### Lack of trust in the vaccine

#### Concerns about vaccine side effects

Some participants expressed fear and concern about vaccine side effects on their health and the health of their fetus or baby as reasons for refusing to get the vaccine. In this regard, one of the participants said: “Due to the unknown side effects of these vaccines and the fact that I was pregnant, in order to protect my fetus, me and my husband thought it would be better not to get vaccinated.“ (P 15) Another participant said: “When the vaccines became available, I was breastfeeding my first child. I did not get the vaccine because I was afraid that it would harm my child.“ (P 5).

Some participants were concerned about the immediate side effects of the vaccine and death. “In one or two months after public vaccination, I heard that there were many side effects and many people died as the result of vaccination. I even know people who had suddenly a heart attack after vaccination. For this reason, I stopped thinking about getting the vaccine altogether. “(P1).

A group of participants was worried about the future side effects of the vaccine. Among others, one of the participants stated that “the vaccines may have side effects happening after a few years. I am worried about my future health. " (P 10).

#### Skepticism on the ineffectiveness of vaccination

Some participants did not believe in the effectiveness of the vaccine. One of them said: “the history of science shows many diseases in the past that could be controlled by vaccination. So, in general, vaccines are effective. But we doubt that these vaccines are effective. The vaccine was produced very fast without enough research I believe. " (P 10) Some of the participants did not trust the effectiveness of the vaccine because even vaccinated people got Corona. One of the participants said: “Not only the number of infected people did not decrease after vaccination, but a large number of those who had been vaccinated were infected in the next peaks of the disease.” (P 4) “In my opinion, Coronavirus disease could be controlled naturally, and each wave naturally immunized a large number of people. According to the statistics, vaccination did not have much effect.“ (P17).

#### Lack of transparency in vaccine-related information and statistics

The distrust in vaccines is rooted partially in the lack of transparent and reliable information and statistics. The unknown nature of the disease significantly impacted the acceptability of COVID-19 vaccine. Many participants stated that the conflicting information broadcast from various national media networks about the benefits and harms of the vaccine disrupted people’s trust in the vaccine. Some of the comments of participants are the following:

“In my opinion, the situation is very strange and everything is suspicious. Channel One national TV showed an interview about the benefits of vaccines last week, but, last night, Channel Four brought a traditional doctor to talk about the harms of vaccines. This contradictory information made me reluctant to go for the vaccine.“ (P 22) “Vaccines that the WHO declare to be completely safe are causing side effects.” (P 23) “There was no information about the side effects of the vaccine at the beginning. The only thing said by the government was to get a vaccine to eliminate the transmission cycle of the disease, which not only did not happen, but the related death rate increased.“(P1).

The unknown nature of the vaccines was also a cause of distrust. A participant stated: “The Ministry of Health did not work very well on vaccines. The authorities did not say what the ingredients of the vaccine are, how they are made and what the side effects are. " (P16).

### Fear of vaccine injection

Some participants were afraid of injections and needles due to previous unpleasant experiences. A participant said: “I generally have a problem with injection. If there was a pill or something else with the effect of the vaccine, I would probably use it. I have been afraid of ampoules, serums, and vaccines since I was a child.” (P17), and another said: “I’m very afraid of injections. My whole family members got vaccinated and I encouraged them to do so. I feel really anxious when the vaccine is getting prepared for me. In my childhood, a wrong diagnosis caused me to have many injections. I’m afraid now” (P 13). “You might think how a nurse can be afraid of syringes. One of the reasons why I did not get vaccinated was being afraid of injections.“ (P10).

### Lack of trust in the government and health officials

Public trust in government and health authorities is critical to vaccination projects. the unavailability of vaccines at the same time as in other countries and subsequent wrong policies, including compulsory vaccination, caused distrust in the government and health authorities. Many healthcare providers believe that to increase the acceptability of the vaccine, the government should not make vaccination compulsory. Below are the statements of a group of respondents.

“Mandatory vaccination is not a right policy when vaccines have not passed the test phase. We still don’t know what will happen in the future to the person who injected the vaccine.“ (P9) “The government makes this decision for every individual (getting vaccinated), but does not bear the financial burden of side effects, hospitalization and medicines. It creates doubts.” (P20).

The unavailability of reliable international vaccines (Pfizer and Moderna, according to participants) caused mistrust. One of them stated: “I wanted to get a certain brand of vaccine, but the government banned its import, and this seemed strange to me and made me doubt about other vaccines. I myself witnessed that many doctors traveled to Turkey or Dubai, got Pfizer vaccine and came back.“(P5) “In my opinion, everyone in the government got Pfizer; while they provided Russian, Chinese and Indian vaccines to the public. Can you come up with a reason why the Pfizer vaccine that the whole world is looking for should not be available for us? " (P16) Another one said: “When it was time to inject my vaccine, they said that the only vaccine we have in stock is Barkat (domestic vaccine). Because I did not trust this vaccine, I refused to get it. When other types of the vaccines became available, the incidence of disease was low so I had no reason to get the vaccine.“ (P17).

### Not feeling the need for a vaccine

Some respondents did not feel the need for a vaccine because of believing in natural immunity, confidence in self-care, and reduced fear of getting infected with Coronavirus.

#### Natural immunity

Many participants believed that they managed to control the coronavirus disease without vaccines and by following health protocols and strengthening the immune system. Also, the perceived risk of coronavirus disease had decreased compared to the first months of the outbreak. In addition, the perceived risk of the complications of the COVID-19 vaccine increased, so people refused to take the vaccine. Many participants stated that in recent months, a large number of medical staff got COVID-19, and their bodies developed antibodies against the disease, so they did not feel the need for vaccination. Some of their statements are as follows:

“At the same time as the vaccination process started, I got infected with coronavirus and my body became immune naturally and I did not go for vaccination anymore on the advice of doctors. " (P1).

#### Confidence in self-care

Some respondents believed they can prevent the disease by following health protocols so they do not need a vaccine. One of them said: “I tried to stay safe by following health protocols such as social distancing particularly from people with symptoms even my family members. So, I did not need to get vaccinated.“ (P 4) “I think the vaccine is good for those who cannot follow health protocols, but I tried to follow them. I have not visited relatives for a long time, and I always wear a mask, I do not go to crowded places. So far, neither myself nor my family, even though my wife has an underlying disease, haven’t got infected " (P27).

#### Decreased fear of getting infected with coronavirus

Among a number of participants, the fear of getting infections and complications has reduced over time. One of them stated:

“In my opinion, the coronavirus is not different from other viruses and it is possible to get infected even though you are vaccinated and follow health protocols. The only way to fight the coronavirus is to boost the immune system. " (P 1) “Now, the fear of vaccine side effects is greater than the fear of death due to coronavirus. (P 5)”.

### The contrast between traditional and modern medicine in their approach to COVID-19 disease

Many participants believed that traditional medicines have fewer side effects than chemical ones, so they refused to get the COVID-19 vaccine. In this regard, a number of participants stated: “One of the reasons why I did not take the vaccine was the advice given by traditional doctors. I really believe in traditional medicine.“ (P7) “By using herbal medicines and following protocols such as social distancing and wearing a mask, I have never caught the disease. I tried to strengthen my immune system with herbal medicines.“ (P 27).

## Discussion

According to the present study, peer recommendations, lack of trust in vaccines, fear of injections, mistrust in the government and health authorities, low perceived risk of disease, and the contrast between traditional and modern medicine about to the disease were the factors related to the refusal of the COVID-19 vaccine among healthcare providers. These results are consistent with previous studies, such as a qualitative study conducted among medical staff in Greece, Romania, France, and Croatia. The study showed hesitation among study participants for acquiring the vaccine. The reasons were mistrust in the government and pharmaceutical companies producing the vaccine, safety, and efficiency issues, instant vaccines’ development, and possible future side effects [22]. A review of 75 articles (divided the factors related to the acceptance of the COVID-19 vaccine into three categories: individual, social, and vaccine-related factors), showed that gender, trust in the government, and concerns about vaccine’s side effects have been declared as the most important factors for the refusal of coronavirus vaccine, which is consistent with the present study’s findings [37]. A systematic review investigating acceptance of the COVID-19 vaccine by Lin et al., showed that fear of vaccine side effects harmed vaccine acceptance [41]. Concerns about vaccine safety and adverse side effects have been the main reasons US healthcare workers to refuse COVID-19 vaccines [13, 22, 23, 42]. Another study in Saudi Arabia obtained similar results [43]. A study exploring vaccine acceptability among medical staff in Ghana (2021) showed that healthcare workers whose relatives have not been infected with COVID-19 were less inclined to inject the vaccines than others [44]. Contrary to these findings, our study showed that vaccine complications in the vaccinated family members, relatives, and colleagues caused medical staff to doubt the vaccine’s safety and efficiency and refuse vaccination. In a study investigating the factors related to the acceptance or refusal of coronavirus vaccine in Iran, having high-risk people in the family and respect for the health rights of others were mentioned as reasons for acquiring the coronavirus vaccine. On the other hand, lack of knowledge about the effectiveness of the vaccine and concerns about the components of vaccines were the main reasons for vaccine refusal [45].

In current study, some participants believed in other prevention methods instead of vaccines. In the study by Bartzek et al. (2021), exploring the attitudes of medical students regarding the COVID-19 vaccine, the participants believed in controlling the epidemic with primary emergency prevention methods and non-pharmacological interventions, such as isolation and quarantine. Therefore, according to them, vaccination was not necessary [46].

Among the participants of the present study, one of the important and influential social factors in the refusal of COVID-19 vaccine was mistrust in the government and health authorities. Healthcare workers would be more likely to accept COVID-19 vaccines if they trusted the government’s efforts to combat the disease. In Bunch’s study (2021) citizens’ trust in government was recognized as essential for trusting the vaccine because it helps overcome the doubts about the safety and effectiveness of vaccines [47]. One of the reasons the study participants gave for refusing the vaccine was the conflicting statistics and information on the quality of vaccines and COVID-19 disease provided by the government, especially in social media. In Garrett’s (2020) study, false information was a major obstacle to the fight against COVID-19 [48]. Some participants addressed mandatory vaccination as a concern. Most of them were against mandatory vaccination. They believed it may have psychological effects and negatively influence compliance with other protective measures. Some healthcare workers expressed concern about the safety and side effects of the COVID-19 vaccines particularly because of the fast development process compared to previous vaccines. For example, in the case of polio, it took a decade or more for the vaccine to be developed and approved [49]. The study by Zuker (2021), which examined the willingness of Swiss healthcare workers to be vaccinated against COVID-19 showed that with the spread of the COVID-19 pandemic, the desire for vaccination and even public demand considerably increased. However, the very fast process of development and approval of vaccines led to mistrust among healthcare staff [50]. Another study indicated that the performance of Iran’s Ministry of Health and Medical Education discouraged acquiring the COVID-19 vaccine. In this study, individual factors were insignificant in refusing the vaccine [51].

Studies by Siegrist (2021), Larsen et al. (2018), and Ozawa et al. showed that mass and social media can play a vital role in strengthening public education about the vaccine. Health policymakers should not let false information about COVID-19 to be spread, especially on social media. Public trust in the COVID-19 vaccine may be built through honest and sincere dialogue between officials and individuals, although this may take considerable time [52–54]. Trust in the healthcare system is positively related to the willingness to receive the COVID-19 vaccine [55–57].

According to the participants of this study, two important sources to obtain information about COVID-19 disease and vaccination were social media and the internet. This result is consistent with the results of Li et al.‘s (2021) study. They found that unwillingness to receive the COVID-19 vaccine was associated with misinformation about vaccines and COVID-19, particularly on social media. Misinformation has been recognized as an obstacle to the fight against COVID-19 [25]. In different studies, healthcare workers addressed the anti-vaccination content of media and its impact on their patients. According to the findings of the studies by Kata et al., Zimmerman et al., and Betsch et al., the general public is increasingly using the internet to research for and share information about vaccines. Analysis of information available on websites and social media shows that negative and sometimes inaccurate content predominates potentially influencing vaccine-related decisions [58–60]. The results of a web-based study that examined the factors influencing the acceptance of the coronavirus vaccine among Iranians showed that risk perception, knowledge of the disease, trust in the health system, attitude towards vaccination, and vaccination literacy were the main drivers of accepting the vaccine [61].

### Research limitation

The study coincided with the hospital requiring the staff to submit proof of vaccination, which caused employee concerns and reduced their willingness to participate in the study.

## Conclusion

This qualitative study explored the factors related to the reluctance of healthcare workers to get vaccinated against COVID-19 in 2022. In total, 28 health personnel who refused to be vaccinated were interviewed. The participants expressed common reasons for vaccine hesitancy, including the fear of vaccines and their side effects, peers’ opinions, not feeling the need for a vaccine, mistrust in the government and health authorities, and the contrast between traditional and modern medicine in their approach to coronavirus disease.

Concerns about the vaccine safety and the side effects were the most influential factors of vaccine hesitancy. Providing reliable and safe vaccines, regularly updating the healthcare workers on vaccine information, and seeking international approvals for domestic vaccines will increase trust and reduce doubts about vaccination. Enhancing personnel’s trust and acceptance can encourage others to get vaccinated. Trust in government officials is associated with the willingness to be vaccinated against COVID-19. Mandatory vaccination can break the trust. It may negatively affect compliance with other protective measures. It is recommended to use positive measures to increase voluntary immunization.

The media has a great influence on the acceptance of COVID-19 vaccine. As this study showed, the dissemination of misinformation increases the doubt among the healthcare staff. Social media must be monitored and managed to prevent the spread of false information. Removing the barriers to accepting vaccination and also providing information about the benefits of the vaccine should be prioritized by health policymakers. Studying the effectiveness of existing vaccines and their benefits in controlling the disease is necessary. Moreover, unrealistic expectations about the effectiveness of vaccines and the subsequent disappointment should be prevented by providing reliable information.

### Electronic supplementary material

Below is the link to the electronic supplementary material.

Supplementary Material 1

## Data Availability

The datasets generated and analyzed during this study are not publicly available because of confidentiality of information but are available from the corresponding author on a reasonable request basis.

## Abbreviations

WHO: World Health Organization
P: Participant
