# Supplementary material for: Why healthcare providers are not vaccinated? A qualitative study during the COVID-19 pandemic in Iran
Source: BMC Prim Care. 2023 Oct 13;24:208. doi: 10.1186/s12875-023-02166-7 (PMC10571274; doi:10.1186/s12875-023-02166-7)
Supplement: Supplementary file 1 — Supplementary Material 1 [file 12875_2023_2166_MOESM1_ESM.docx]

Interview questions

1. In your opinion, what are the ways to prevent the spread of corona disease? Which one is the most effective?
2. As a healthcare staff, do you advise people to get vaccinated?
3. Please explain your reasons for refusing corona vaccine.
4. If other vaccines were available, would you refuse the vaccine?
5. Do you think vaccine can prevent corona disease? To what extent?
6. What is your opinion about the side effects of the vaccine? Do you think the side effects are more than the benefits of vaccination?
7. From whom do you get advice about corona vaccine?
8. What are your reliable sources of vaccine information?
9. Do you think that sufficient and quality information about vaccines has been provided by the government? (e.g., the type of vaccines available and the effectiveness of vaccines)
10. What do you think about mandatory vaccination?
11. What actions by the government can encourage vaccination?
12. In your opinion, in what ways, trust in vaccination can be built?
